# *GsCML27*, a Gene Encoding a Calcium-Binding Ef-Hand Protein from *Glycine soja*, Plays Differential Roles in Plant Responses to Bicarbonate, Salt and Osmotic Stresses

**DOI:** 10.1371/journal.pone.0141888

**Published:** 2015-11-09

**Authors:** Chao Chen, Xiaoli Sun, Huizi Duanmu, Dan Zhu, Yang Yu, Lei Cao, Ailin Liu, Bowei Jia, Jialei Xiao, Yanming Zhu

**Affiliations:** 1 Key Laboratory of Agricultural Biological Functional Genes, Northeast Agricultural University, Harbin, P.R. China; 2 Agronomy College, Heilongjiang Bayi Agricultural University, Daqing, P.R. China; 3 College of Life Science, Qingdao Agricultural University, Qingdao, P.R. China; Institute of Genetics and Developmental Biology, Chinese Academy of Sciences, CHINA

## Abstract

Calcium, as the most widely accepted messenger, plays an important role in plant stress responses through calcium-dependent signaling pathways. The calmodulin-like family genes (*CMLs*) encode Ca^2+^ sensors and function in signaling transduction in response to environmental stimuli. However, until now, the function of plant CML proteins, especially soybean CMLs, is largely unknown. Here, we isolated a *Glycine soja* CML protein GsCML27, with four conserved EF-hands domains, and identified it as a calcium-binding protein through far-UV CD spectroscopy. We further found that expression of *GsCML27* was induced by bicarbonate, salt and osmotic stresses. Interestingly, ectopic expression of *GsCML27* in *Arabidopsis* enhanced plant tolerance to bicarbonate stress, but decreased the salt and osmotic tolerance during the seed germination and early growth stages. Furthermore, we found that ectopic expression of *GsCML27* decreases salt tolerance through modifying both the cellular ionic (Na^+^, K^+^) content and the osmotic stress regulation. *GsCML27* ectopic expression also decreased the expression levels of osmotic stress-responsive genes. Moreover, we also showed that GsCML27 localized in the whole cell, including cytoplasm, plasma membrane and nucleus in *Arabidopsis* protoplasts and onion epidermal cells, and displayed high expression in roots and embryos. Together, these data present evidence that GsCML27 as a Ca^2+^-binding EF-hand protein plays a role in plant responses to bicarbonate, salt and osmotic stresses.

## Introduction

Crop growth and productivity is adversely affected by environmental challenges, such as salinity, alkalinity and osmotic stresses. Sodium bicarbonate stress, including HCO_3_^-^, CO_3_^2-^, Na^+^ and high pH induces the disorder of intracellular pH and hyperosmotic stress in plant cells, and elicits adverse effects plant on growth [1–5]. With the development of transcriptional profiling approaches, recent studies have identified the bicarbonate responsive genes in *Tamarix androssowii* by using gene chip analysis [6], in *Glycine soja* by using transcriptome analysis [7] and in *tomato* by using iTRAQ-based analysis [8]. A handful of researches also characterized some genes involved in plant tolerance to bicarbonate stress. For example, *GsTIFY10* have been identified as a positive regulator of plant tolerance to bicarbonate stress [9], and overexpression of *H* ^*+*^ *-Ppase* improves saline-alkaline tolerance in *Arabidopsis* [10]. However, the physiological and biochemical mechanism of plant bicarbonate stress responses is still unclear.

In addition to bicarbonate stress, neutral salt and osmotic stresses also induce a wide range of complex cellular and physiological changes in plants. The signaling of salt and osmotic stresses include ionic and osmotic homeostasis signaling [11–14], as well as signaling to coordinate cell division and expansion suitable for the particular stress conditions [15, 16]. Many researches also demonstrated the crosstalk of salt stress and osmotic stress [17–19]. For example, expression of the osmotic stress-responsive genes *RD22*, *P5CS* and *COR47* can also be induced by salt stress [14, 20–22]. However, several studies also revealed the differences among plant responses to bicarbonate stress, neutral salt and osmotic stresses [5, 23]. Therefore, besides the common molecular basis and signal transduction pathway, it is also important to discover how plant differentially perceive and transmit the signals under these different stresses.

Calcium (Ca^2+^), as the most widely accepted second massager, plays an important role in plant stress responses[24–26], and cytoplasmic Ca^2+^ signal is recognized by Ca^2+^ sensors [27–29]. Ca^2+^ sensors can be broadly divided into four groups, incuding calmodulins (CaM), calmodulin-like proteins (CMLs), calcium dependent protein kinases (CDPKs) and calcineurin B-like proteins (CBLs) [30, 31]. CMLs share at least 16% amino acid identity with CaMs, and are defined by the presence of two to six EF-hands motifs, and EF-hand motif is a helix-loop-helix structure that can bind a single Ca^2+^ ion [32]. There are 50 CML genes in *Arabidopsis*, and expression analysis suggests that they can be induced by various environmental stresses [33, 34]. Among them, *CML24* is identified as a salt responsive gene, and its overexpression *Arabidopsis* lines are more tolerant to various ions including Co^2+^, Zn^2+^ and Mg^2+^ [35]. *CML18* plays a role in salinity tolerance through direct interaction with the Na^+^/H^+^ antiporter NHX1 [36]. Furthermore, *CML9* is suggested to be a negative regulator of ABA (Abscisic acid) -dependent salinity tolerance [37]. In addition, *CML37*, *CML38* and *CML39* also respond to a variety of environmental stimuli [38]. However, functional evidence of soybean CMLs different environmental stresses is still limited.

In this study, we isolated a CML protein GsCML27 from the salt-alkaline resistant wild soybean *Glycine soja* (07256) [39]. GsCML27 contains four conserved calcium-binding EF-hand motifs and showed Ca^2+^ binding affinity in vitro. We demonstrated the induced expression of *GsCML27* in response to bicarbonate, salt and osmotic stresses. What is interesting is *GsCML27* ectopic expression in *Arabidopsis* enhanced plant tolerance to bicarbonate stress, but decreased salt and osmotic tolerance.

## Results

### Cloning and sequence analysis of *GsCML27*

In our previous study [40], *GsCML27* was identified as a putative bicarbonate stress (50mM NaHCO_3_, pH8.5) responsive gene, by transcriptome sequencing data of the wild type soybean *Glycine soja* G07256 (S1 Fig). In this study, the full-length CDS of *GsCML27* was obtained by homologous cloning with gene-specific primers designed according to the transcript sequence of *Glycine max* homolog (Glyma08g05810). *GsCML27* contains an open reading frame of 543 bp that encoded a protein of 181 residues with a predicted molecular weight of 19.7 kDa and an isoelectric point of 4.32.

**Fig 1:**
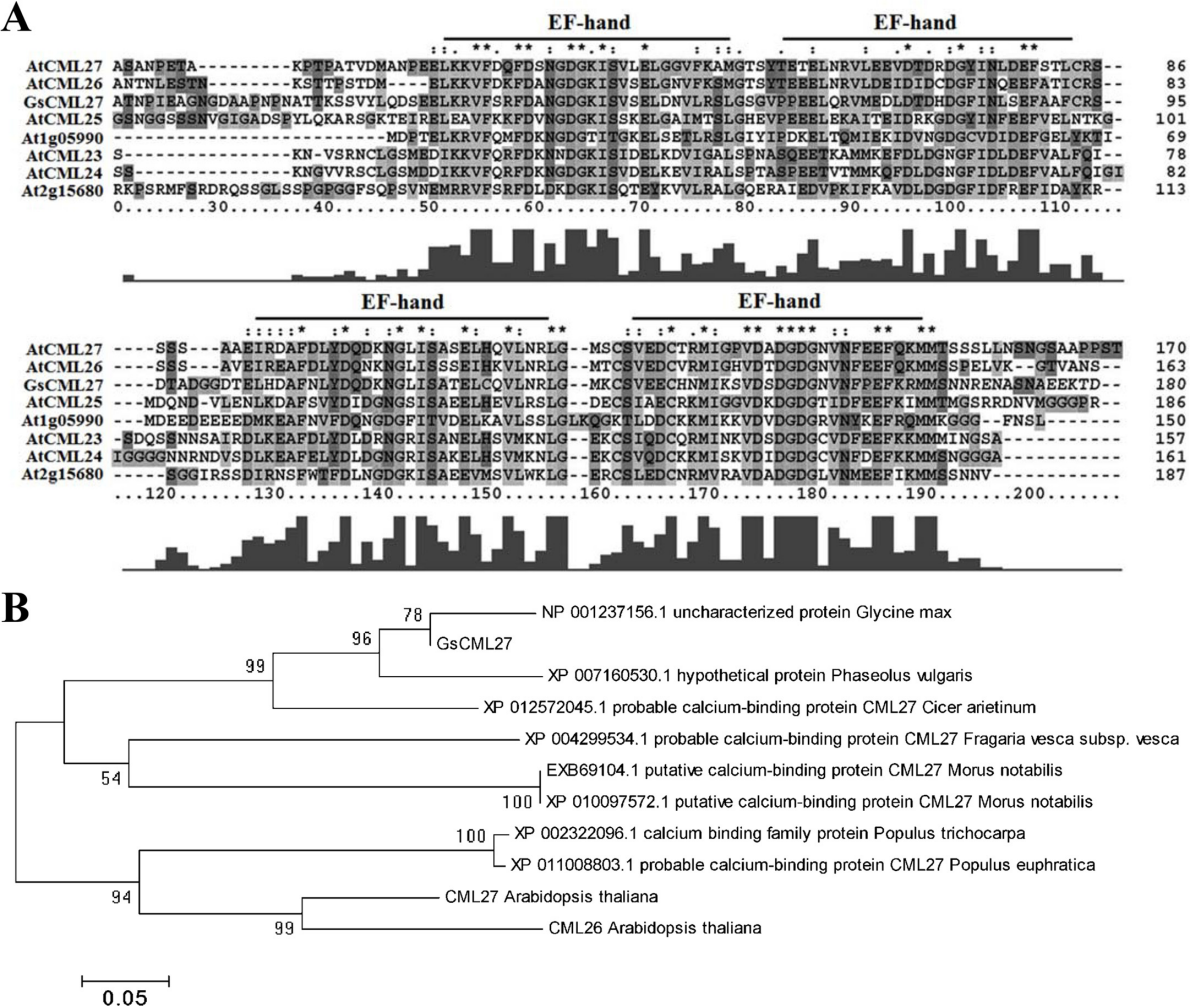
Sequence analysis of *GsCML27*. **A**. Multiple sequence alignment of the full-length amino acid sequences of *GsCML27* with homologous *CMLs* from *Arabidopsis*. Sequences were aligned using MEGA 5.0. **B**. The phylogenetic relationships between *GsCML27* and homologous protein kinases with the conserved amino acid sequences. The phylogenetic tree was constructed using MEGA 5.0.

Protein sequence analysis showed GsCML27 contains four highly conserved calcium-binding EF-hand domains, and shared 47.8% to 61.7% sequence similarity with *Arabidopsis* CML family proteins (http://www.phytozome.org/) [41, 42] (Fig 1A). Among them, GsCML27 displayed the highest amino acid sequence similarity (61.7%) with AtCML27. Phylogenetic analysis showed that GsCML27 was clustered with other CML27-like proteins, such as *Cicer arietinum*, *Fragaria vesca subsp*. *Vesca* and *Morus notabilis* (Fig 1B). These results indicated that *GsCML27* might be one of CML family members.

### Circular dichroism (CD) spectroscopy and Subcellular localization of GsCML27

**Fig 2:**
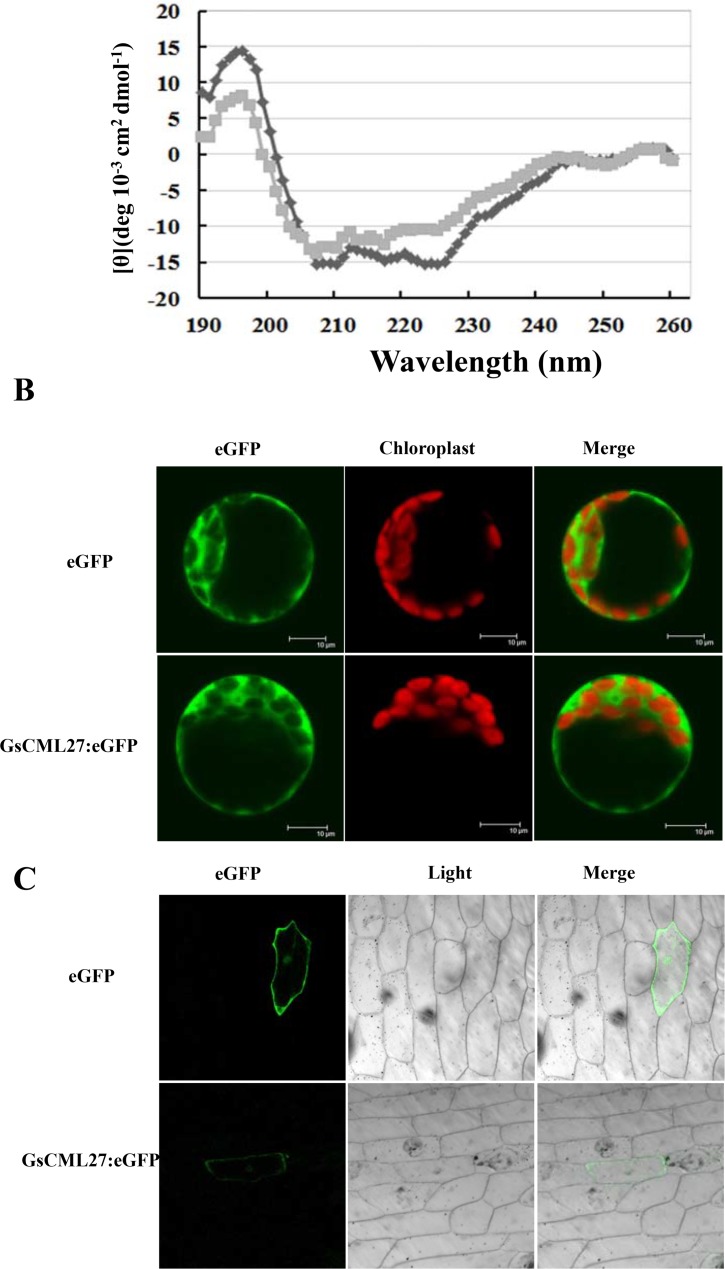
Circular dichroism (CD) spectroscopy assay and subcellular localization of GsCML27. **A**. Ca^2+^-induced conformational changes of the GsCML27. Ca^2+^-induced conformational changes of the GsCML27 were monitored by far-UV CD spectroscopy. Spectra were collected on samples in 5 mM Tris-HCl, pH 6.9, in the presence of either 5 mM CaCl_2_ or 5 mM EGTA. Results of all CD measurements are expressed as mean molar ellipticity [θ]. **B**. Subcellular localization of GsCML27 protein in *Arabidopsis* protoplasts. eGFP-tagged GsCML27 fusion protein and eGFP alone protein were transiently expressed in the protoplasts prepared from 3-week-old *Arabidopsis* leaves and checked the eGFP signal by using a confocal laser-scanning microscope. The eGFP or the GsCML27-eGFP fusion protein examined under fluorescent-field illumination to examine GFP fluorescence (left); Fluorescent-field illumination for chlorophyll fluorescence was used to examine chlorophyll fluorescence (middle), followed by the confocal microscopy for an overlay of GFP and chlorophyll fluorescent illumination (right). **C**. Subcellular localization of GsCML27 protein in onion epidermal cells. eGFP-tagged GsCML27 fusion protein and eGFP fluorescence, bright field and an overlay of bright and fluorescent illumination are shown.

The four EF-hand motifs in GsCML27 indicated potential binding affinity to Ca^2+^. We expressed and purified the GsCML27-His fusion protein from *E*.*coli* BL21. Far-UV CD spectroscopy was used to assess the impact of Ca^2+^ binding on the conformation of GsCML27. As shown in Fig 2A, The addition of CaCl_2_ to GsCML27 protein results in a slight increase in the 207–244 nm range suggesting a Ca^2+^-induced increase in α-helical content. Deconvolution of the data predicts a 55% increase in helical content from 10% to 65%. These results suggested that GsCML27 could bind Ca^2+^ and might undergo a Ca^2+^-dependent conformational change [43, 44].

In order to investigate the sub-cellular localization of GsCML27, the CDS region of *GsCML27* was in-frame fused with the enhanced green fluorescence protein (eGFP) to generate 35S::GsCML27-eGFP. The eGFP and GsCML27-eGFP fused protein were transiently expressed in *Arabidopsis* protoplasts respectively. The green fluorescence was observed by confocal scanning. The eGFP alone was used as control and result showed that eGFP localized to the entire *Arabidopsis* protoplast cell. Confocal imaging showed that GsCML27-eGFP protein was expressed in the whole protoplast cell, including cytoplasm, plasma membrane and nucleus (Fig 2B).

To further confirm the subcellular localization of GsCML27 in protoplasts. The plasmids eGFP and GsCML27-eGFP were transiently expressed in onion epidermal cells. The fluorescence of eGFP alone appeared through the whole cell. GsCML27-eGFP was also observed in the whole protoplast cell, which is consistent with results from protoplast cells (Fig 2C). Taken together, these results suggested that GsCML27 encodes a ubiquitously expressed calcium-binding protein.

### Expression profiles of *GsCML27* in *Glycine soja*

**Fig 3:**
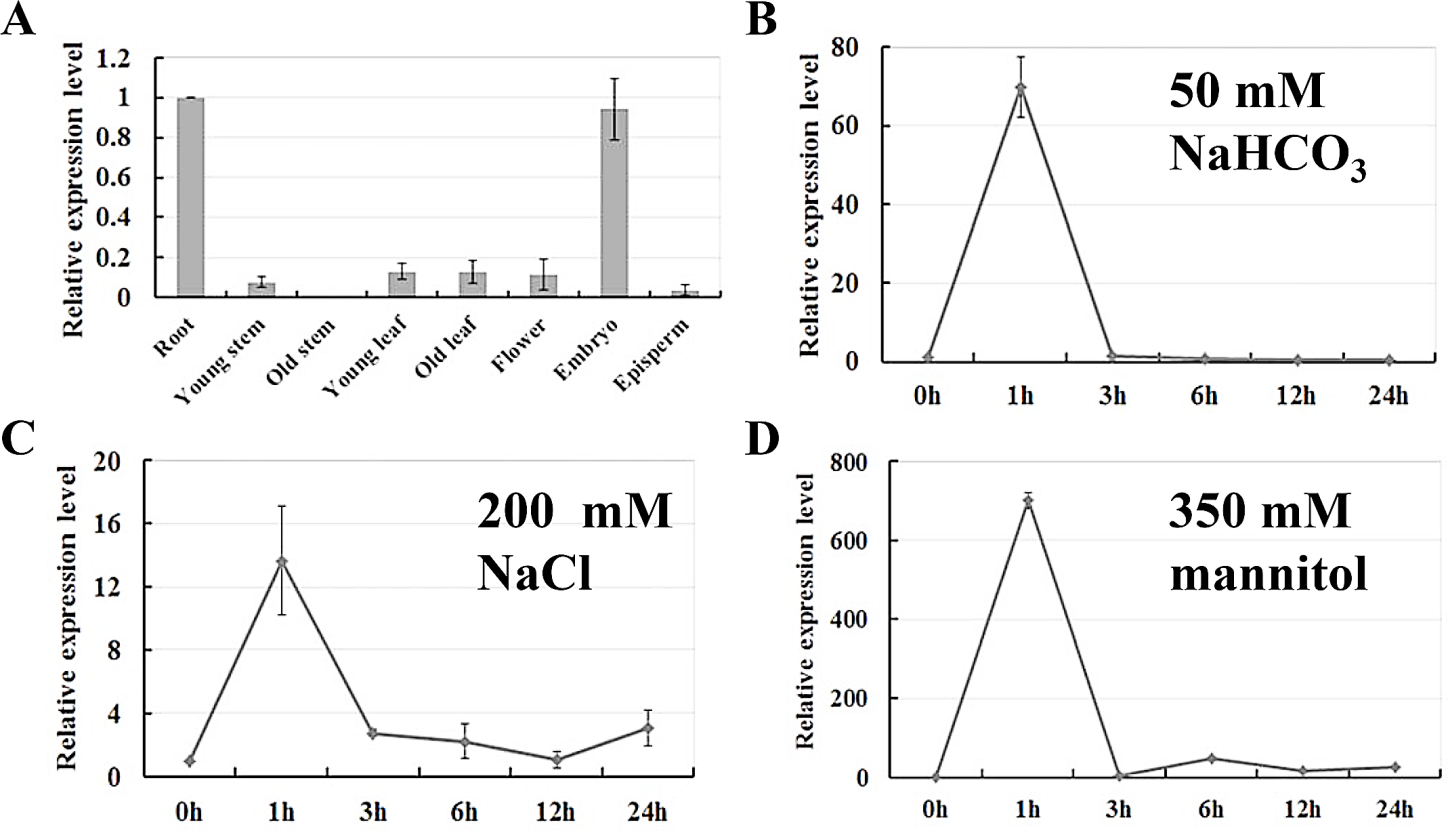
Expression profiles of *GsCML27* in *Glycine soja*. **A**. Tissue specific expression of *GsCML27* in *Glycine soja*. Total RNA was extracted from root, young stem, old stem, young leaf, old leaf, flower, embryo and episperm from 5-week-old soil-grown Glycine soja, and relative expression levels were determined by quantitative RT-PCR using *GADPH* as an internal control. **B**. Total RNA was extracted from roots of 3-week-old *Glycine soja* seedlings treated with 50 mM NaHCO_3_, 200 mM NaCl (salt stress) and 350 mM mannitol (osmotic stress) treatments, respectively. Relative transcript levels were determined by quantitative RT-PCR and *GAPDH* were used as internal controls. The mean value from three fully independent biological repeats and three technical repeats is shown.

To characterize the spatial expression pattern of *GsCML27* in *Glycine soja*, the expression levels of *GsCML27* in different tissues were detected by quantitative RT-PCR analysis. The results showed that *GsCML27* was expressed in most of the tissues in this study. The highest expression of *GsCML27* was observed in roots and embryos, and little expression was obtained in old stems (Fig 3A), indicating tissue specificity of *GsCML27* expression in *Glycine soja*.

In order to investigate the potential role of GsCML27 in stress responses, we then verified the bicarbonate stress induced expression of *GsCML27* in *Glycine soja*. Consistent with the RNA-seq data, the quantitative RT-PCR results confirmed that expression of *GsCML27* increased and reached a maximum point at 1 h (about 70 folds) under bicarbonate stress (Fig 3B). Then *GsCML27* transcripts decreased to the basal level after 3 h, indicating *GsCML27* responded to bicarbonate stress at an early stage.

Considering the fact that ion poison and osmotic stress always occurred simultaneously with bicarbonate stress [45, 46], we also determined the expression profiles of *GsCML27* under 200 mM NaCl (salt stress) and 350 mM mannitol (osmotic stress) treatments (Fig 3C and 3D). As expected, *GsCML27* expression was also induced by both salt and osmotic stresses, and displayed similar patterns compared to bicarbonate stress. Notably, the increase of *GsCML27* expression under osmotic stress (about 700 folds) was obviously greater than that under salt stress (about 14 folds). Taken together, these results indicated that *GsCML27* might be involved in plant responses to bicarbonate, salt and osmotic stresses.

### The opposite roles of *GsCML27* in response to bicarbonate versus salt/osmotic stresses

**Fig 4:**
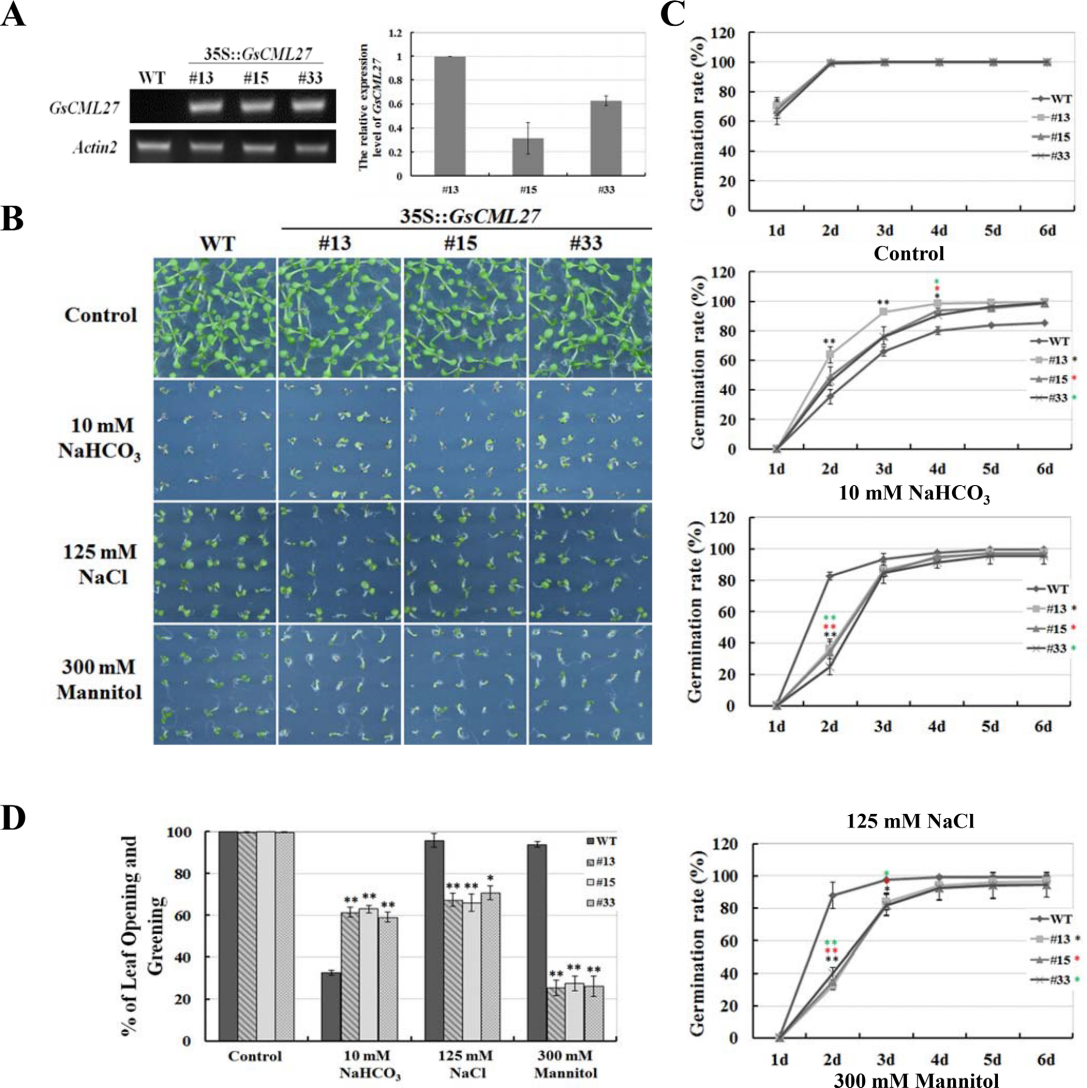
Ectopic expression of *GsCML27* in *Arabidopsis* enhanced bicarbonate tolerance, but decreased salt and osmotic tolerance. **A**. Semi-quantitative RT-PCR and quantitative RT-PCR analysis of *GsCML27* expressions in WT and three ectopic expression lines. **B**. Growth performance of WT and ectopic expression seedlings on 1/2 MS medium without or with 10 mM NaHCO_3_, 125 mM NaCl or 300 mM mannitol. Photographs were taken 7 days after stratification. **C**. Seed germination rates of WT and ectopic expression lines under stresses. Germination was recorded daily up to 6 days. **D**. Seedlings with open and green leaves was recorded 7 days after stratification. All of the values represent the means of three fully independent biological replicates; error bars indicate the SD. *P < 0.05 and **P < 0.01 by Student’s t-test.

In order to determine whether *GsCML27* is involved in plant responses to bicarbonate, salt and osmotic stresses, transgenic *Arabidopsis* plants were generated by overexpressing *GsCML27* under the control of strong constitutive CaMV35S promoter. Three independent homozygous transgenic lines (#13, #15 and #33) were obtained and the transcript abundance of *GsCML27* was verified by semi-quantitative RT-PCR and quantitative RT-PCR analysis (Fig 4A).

We firstly performed the plate germination assays to determine the stress tolerance of WT and *GsCML27* ectopic expression *Arabidopsis* lines (Fig 4B). Under standard culture conditions, *GsCML27* ectopic expression lines exhibited similar seed germination and early seedling growth with WT, suggesting that *GsCML27* did not affect seed germination and early seedling development (Fig 4C). However, under 10 mM NaHCO_3_ stress treatment, *GsCML27* ectopic expression lines displayed higher seed germination rates (Fig 4C) and more seedlings with open and green leaves (Fig 4D) than WT. Unexpectedly, on 1/2 MS medium containing 125 mM NaCl or 300 mM mannitol, WT exhibited better at seed germination (Fig 4C) and seedlings growth (Fig 4D), as evidenced by higher seed germination rates and more seedlings with open and green leaves than ectopic expression lines. These results demonstrated that *GsCML27* enhanced plant tolerance to bicarbonate stress, but decreased the salt and osmotic tolerance.

### *GsCML27* influences plant responses to K^+^ ionic stress

Salt stress affects plant growth and development mainly in two ways: ionic poison and osmotic stress. As described above, we have demonstrated *GsCML27* participated in the osmotic regulation in plant cells, showing decreased tolerance to 300 mM mannitol treatment. Hence, we then explored whether *GsCML27* responded to different ionic stresses.

**Fig 5:**
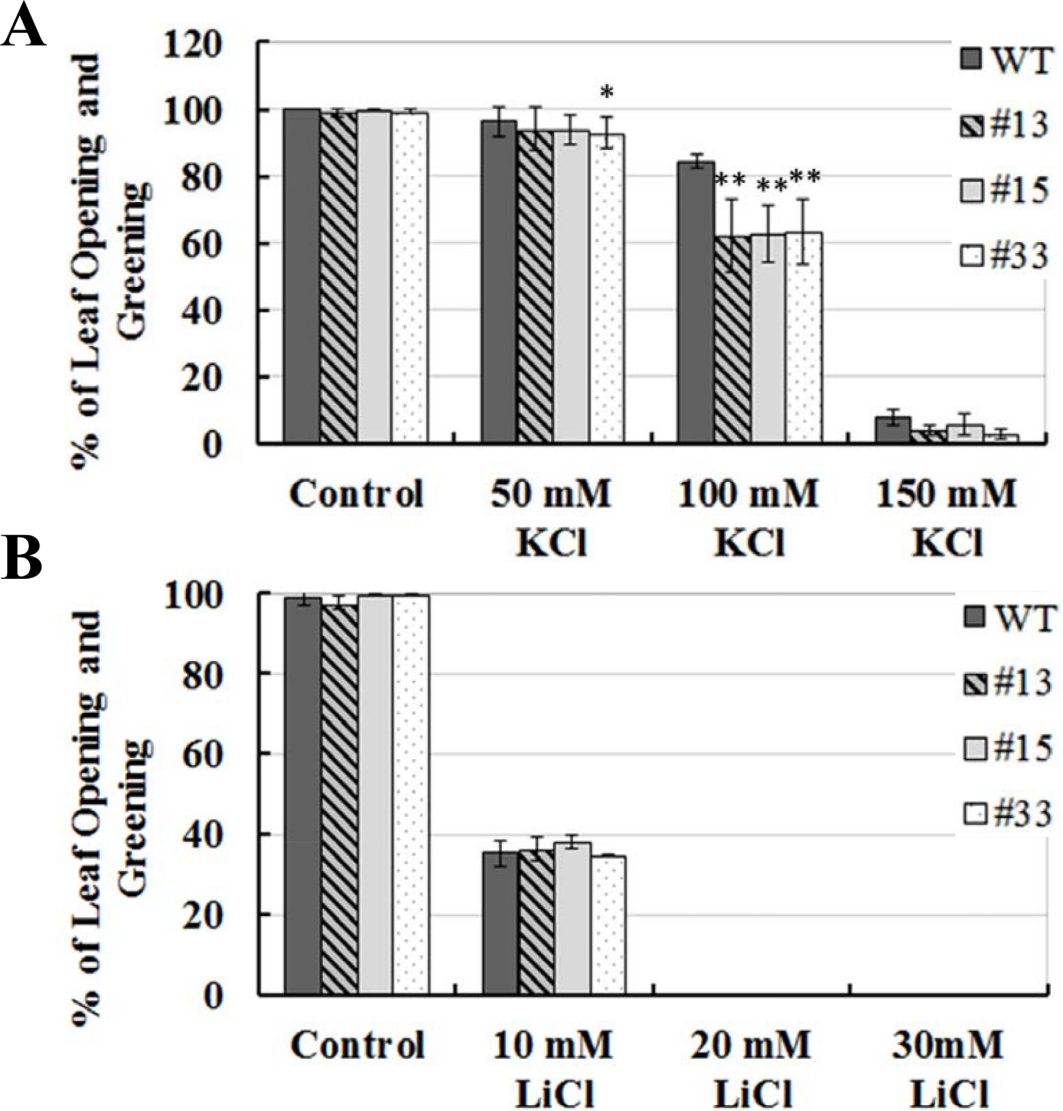
Ectopic expression of *GsCML27* in *Arabidopsis* influences plant by modifying cellular ionic content. **A**. Growth performance of WT and ectopic expression seedlings on 1/2 MS medium with 50, 100, 150 mM KCl. Seedlings with open and green leaves was recorded 7 days after stratification. **B**. Growth performance of WT and ectopic expression seedlings on 1/2 MS medium with 10, 20, 30 mM LiCl. Seedlings with open and green leaves was recorded 7 days after stratification. Data shown represent the means (±SE) of three independent experiments. *P < 0.05 and **P < 0.01 by Student’s t-test.

As shown in S2 Fig, in the presence of different KCl concentration gradients (50, 100, 150 mM KCl), *GsCML27* ectopic expression lines displayed no differences in the seed germination rates from WT. However, under 100 mM KCl treatment, ectopic expression lines showed much lower percentages of seedlings with open and green leaves on the 7th day after germination (Fig 5A). Furthermore, no significant differences in seed germination (S2 Fig) and early seedling growth (Fig 5B) were observed between WT and ectopic expression lines under different LiCl (10, 20, 30 mM) concentration gradients. These results indicated that *GsCML27* regulated plant salt tolerance by modifying both cellular ionic content (mainly Na^+^, K^+^) and osmotic regulation.

### *GsCML27* regulated the expression levels of osmotic stress responsive marker genes

**Fig 6:**
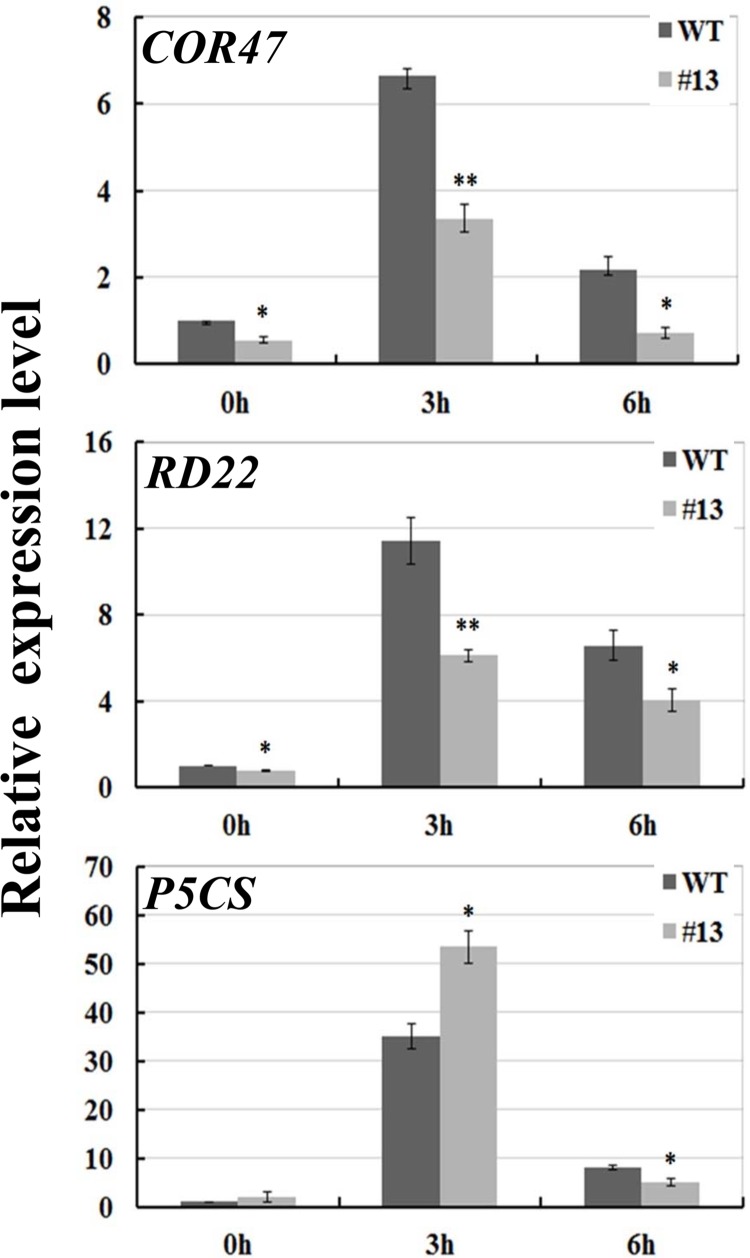
*GsCML27* ectopic expression altered expression patterns of a set of osmotic stresses signal related genes. Expression patterns of osmotic stress related genes. Relative expression levels were determined by quantitative RT-PCR using *ACTIN2* as an internal control. All of the values represent the means of three fully independent biological replicates; error bars indicate the SD. *P < 0.05 and **P < 0.01 by Student’s t-test.

The analysis of some stress-inducible marker genes is a hallmark of stress adaptation in plants [47]. Considering the huge changes of *GsCML27* under osmotic stress, we then examined the expression patterns of some osmotic stress induced genes, including *COR47*, *RD22* and *P5CS*. As shown in Fig 6, their expression levels were obviously induced by osmotic stress. However, their expression levels were significantly down-regulated in *GsCML27* ectopic expression lines compared to WT, except for *P5CS* at 3 h. Therefore, these results suggested that *GsCML27* regulated the expression levels of osmotic stress responsive marker genes.

## Discussion

Plants have evolved a diversity of unique proteins containing evolutionarily conserved EF-hand motifs to bind Ca^2+^ [48, 49]. Among them, *CML* (*CAM-like*) family, proteins are mostly composed of 2 to 6 EF-hands, and play important roles in plant growth, development and environmental stimuli stress responses [33]. Indeed, studies have revealed the important function of *CML42* during cell branching in trichomes [50]. *CML18* was involved in salinity tolerance and *CML9* knockout enhanced plant tolerance to both salinity and drought stresses [36, 51]. However, most members of *CMLs* family are functionally uncharacterized. In this study, we isolated and characterized *GsCML27*, a calcium-binding EF-hand protein from *Glycine soja*.

Studies have revealed both *GsTIFY10* and *AtTIFY10* have positive function in alkaline responses [52, 53]. Overexpression of *GhWRKY25* in *Nicotiana benthamiana* enhances plant tolerance to salt stress [54], overexpression of *WRKY25* is also sufficient to increase *Arabidopsis* NaCl tolerance [55]. In this study, GsCML27 shared the highest amino acid similarity with AtCML27 (61.7%) among the 50 CML proteins in reference plant *Arabidopsis* [33]. Similar to AtCML27 and other *Arabidopsis* CMLs proteins, GsCML27 contains four conserved EF-hand motifs (Fig 1A). So we indicate that *AtCML27* may have similar function with *GsCML27*. Studies have shown *Arabidopsis* CML8 [56] and CML24 [57] have Ca^2+^-binding ability so that they can take part in signaling transduction in response to diverse stimuli. As expected, we also verified the Ca^2+^-binding property of GsCML27 protein through the far-UV CD spectroscopy (Fig 2A).

The majority of CMLs are predicted to be located in cytosolic or nuclear in *Arabidopsis* [33]. *CML42*, a calcium sensor, localized to both cytosol and nucleus responses to spodoptera herbivory and abiotic stresses[58]. To prove the GsCML27 might correlate with the spatial pattern of Ca^2+^ elevation, we observed the localization of GsCML27. As a result, we observed GsCML27 protein are localized in the whole cell, including cytoplasm, plasma membrane and nucleus (Fig 2B and 2C). So it can be assumed that GsCML27 can bind Ca^2+^ and undergo conformational changes to bind target proteins in the whole cell.

A handful of research have found that *CMLs* expression were greatly induced by environmental stresses such as drought, salt and osmotic stress [36–38, 59]. For example, studies have revealed *CML37*, *CML38* and *CML39* temporal response to drought, salt and osmotic stresses [60], and AtCML9 played essential roles in modulating responses to salt stress [51]. In the present research, we also found the induced expression of *GsCML27* under salt and osmotic stresses (Fig 3C and 3D). It is noteworthy that *GsCML27* expression was also strongly induced by bicarbonate stress, as supported by the transcriptome data (S1 Fig) and quantitative RT-PCR assays (Fig 3B). Interestingly, based on the RNA-seq data, we further identified a group of genes co-expressed with *GsCML27* (S2 Table). These genes were found to be involved in plant stress responses, including three alkaline responsive genes (*GsJAZ2*, *GsTIFY10a*, *GsTIFY11b*) [52, 61, 62] and nine salt/drought/osmotic responsive genes (for example: calmodulin binding protein 25, transcription factor MYB44, myb domain protein 78) [63–65]. Overall, these data suggested that *GsCML27* may extensively regulator plant responses to environmental stresses.

As expected, we further demonstrated the important function of GsCML27 in stress responses. However, intriguingly, *GsCML27* ectopic expression promoted seed germination in response to bicarbonate stress, but inhibited seed germination under salt and osmotic stresses during the plate germination assays (Fig 4). Similar phenomenon were also found for CBL1, which serves as a positive regulator of the salt and drought signaling pathways and as a negative regulator of the cold response pathway in *Arabidopsis* [66]. One explanation for this fact is that the specificity and complexity of *GsCML27* function in different stress signaling pathways, and studies also suggest that different pathways may share common components that serve as crosstalk nodes [67].

Salinity exerts negative impact mainly by disrupting the cell ionic and osmotic equilibrium [68, 69]. The decreased tolerance under osmotic stress suggested that *GsCML27* was involved in osmotic regulation under salt stress. To explore whether *GsCML27* affected salt tolerance only by osmotic regulation or by both ionic and osmotic regulation, we carried out the plate germination assays under KCl and LiCl treatments (Fig 5 and S2 Fig). Our results revealed that besides osmotic regulation, *GsCML27* might also affect plant tolerance by modifying intracellular Na^+^/K^+^ content [18, 70]. In line with our studies, *CML24* was found to participate in plant responses to ion stress [35]. AtCML18 interacted with AtNHX1 (a vacuolar Na^+^/H^+^ antiporter) in a Ca^2+^/pH-dependent manner, and repressed the Na^+^/H^+^ exchange activity [36]. The expression levels of *CML37*, *CML38* and *CML39* also induced by osmotic stress [38]. Moreover, we showed that *GsCML27* ectopic expression also down-regulated expression of osmotic responsive genes, such as *P5CS*, *COR47*, and *RD22* (Fig 6). It is not known, however, how important the mechanisms under salt stress will be an important area for further studies.

Another intriguing things is that no obvious phenotypes between WT and *GsCML27* ectopic expression lines was found during the root length assays at the seedling stage (data not show). One possible reason is *GsCML27* mainly function at the seed germination and/or early seedlings stages. Our studies also found that *GsCML27* displayed high transcript level in the wild soybean seeds, and the expression of *GsCML27* significantly decreased with the seed germination and seedling growth (S3 Fig). Additionally, in *Arabidopsis*, *AtEXP2* only confered salt and osmotic stress tolerance at seeding germination stages [71], and overexpression of *AtWRKY30* also enhanced abiotic stress tolerance during early growth stages [72].

In conclusion, GsCML27 is a positive regulator of plant tolerance to bicarbonate stress, but a negative regulator of salt or osmotic stresses during early growth stages. And ectopic expression of *GsCML27* may influence plant salt tolerance by ion content (Na+/K+) and mainly osmotic stress. However, for the future studies, we will focus on the exact mechanism by which *GsCML27* responses to bicarbonate, salt or osmotic stress, and explore the relationship between bicarbonate and salt/osmotic stresses.

## Materials and Methods

### Plant materials, growth conditions and stress treatments

Seeds of *Glycine soja* (07256) were acquired from Jilin Academy of Agricultural Sciences (Changchun, China). The seeds were treated with 98% sulfuric acid for 15 min, washed with sterilized water, germinated in a dark culture room, and then grew in 1/4 Hoagland solution at 24–26°C and a light regime of 16 h light/8 h dark [52]. For gene expression analysis, the 3-week-old seedlings were transferred into 1/4 Hoagland’s solution containing either 50 mM NaHCO_3_ (for bicarbonate treatment), or 200 mM NaCl (for salt treatment), or 350 mM mannitol (for osmotic treatment), respectively. Equal amounts of roots were sampled at 0 h, 1 h, 3 h, 6 h and 12 h time points after treatments and the samples were stored at -80°Cfor RNA extraction.

The *Arabidopsis thaliana* (Columbia) were grown in a greenhouse under controlled environmental conditions (21–23°C, 100 μmol photons m^-2^s^-1^, 60% relative humidity, 16 h light/8 h dark cycles). For the expression analysis of osmotic responsive marker genes, the 3-day-old wild-type (WT) and *GsCML27* ectopic expression transgenic (line #13) plants were harvested from 1/2 MS liquid plates containing 300 mM mannitol.

### Isolation and sequence characterization of GsCML27

The CDS region of GsCML27 was obtained by using homologous cloning. Briefly, total RNA was isolated from *Glycine soja* seedling roots by using an RNeasy Plant Mini Kit (Qiagen, Valencia, CA, USA), and then the cDNA was generated using the SuperScriptTM III Reverse Transcriptase Kit (Invitrogen,Carlsbad, CA, USA). Gene specific primers (FW 5’-CTGTTGAAAGCGATAGCAATGGC-3’ and RV 5’-CTATAAAATTCA AATCCAAAGGGCC-3’) were designed according to the corresponding gene sequence from *Glycine max* (Glyma08g05810) to clone the full-length CDS of GsCML27. The PCR products were cloned into the pGEM-T vector (Promega, Madison, WI) and subjected to sequencing. Sequence alignment and phylogenetic analysis were carried out by using MEGA 5.0 [73].

### GsCML27 protein expression and circular dichroism (CD) spectroscopy assay

The full-length CDS of *GsCML27* was amplified by PCR with a forward primer containing a *KpnI* site 5’-GGGGTACCATGGCCACGAATCCAATCG-3’ and a reverse primer containing a *SalI* site 5’-ACGCGTCGACATCTGTTTTTTCTTCAGCATTG-3’. The PCR products were cloned into the pET-32b vector (Promega, Madison, WI), Where *GsCML27* was fused to the C/N-terminus of His-tag. The recombinant plasmid was transformed into *E*. *coil* BL21. Protein expression was induced with 1 mM isopropyl thiogalactopyranoside (IPTG) at 37°C. Bacterial cells were harvested after IPTG induction for 6 h by centrifuging the culture at 5000 g for 8 min. The cells was resuspended cells in 8 ml 1/5 Native Binding Buffer (2.5 M NaCl, 250 mM NaH_2_PO_4_, pH 8.0), using a sonicator to broken bacteria cells, sonicate the solution on ice using six 15-second bursts at high intensity with a 20-second cooling period between each burst, centrifuge at 12000 × g for 15 minutes to pellet the cellular debris and transfer the supernatant to a fresh tube. For recombinant protein purification, the procedure followed ProBond^TM^ Purification System (Invitrogen, Carlsbad, CA, USA). Added 8 ml of supernatant under native conditions to a prepared Purification Column, binding 30–60 minutes and then settle the resin by low speed centrifugation (800 × g). Washed with 8 ml Native Wash Buffer (30 ml, 1 × Native Binding Buffer and 100 μl 3M Imidazole, PH 8.0) three more time, and elute the protein with 8 ml Native Elution Buffer (13.75 ml, 1 × Native Binding Buffer and 1.25 ml 3M Imidazole, PH 8.0). Collected fractions and analyzed with SDS-PAGE.

Far-UV CD spectra of GsCML27 assay was carried out according to the method described previous [50, 74, 75]. Far-UV CD spectra were acquired from 190 to 260 nm on a rapid scanning monochromator fitted with a CD module (J-815, Jasco, Japan), using a 0.1 mm path length cylindrical quartz cuvette at 25°C. Spectra were collected on samples in 5 mM Tris-HCl, pH 6.9, in the presence of either 5 mM CaCl_2_ or 5 mM EGTA. Results of all CD measurements are expressed as mean molar ellipticity [θ] = θ*100/(ncl), where n is the mean the number of amino acids of the protein, c is the protein concentration (mg/cm^3^), and l is the path length (cm).

### Quantitative RT-PCR

Quantitative RT-PCR was used to analyze the spatial expression characteristic of *GsCML27 soja* under 50 mM NaHCO_3_, or 200 mM NaCl, or 350 mM mannitol stresses in *Glycine soja* and expression profiles of *GsCML27* and stress responsive marker genes (*P5CS*, *COR47*, and *RD22*) under osmotic stresses in ectopic expression lines. Total RNA was isolated using an RNeasy Plant Mini Kit (Qiagen, Valencia, CA, USA), and the cDNA was generated using the SuperScript^TM^ III Reverse Transcriptase kit (Invitrogen, Carlsbad, CA, USA). Quantitative RT-PCR was performed by using the SYBR Green Master Mix on an ABI 7500 sequence detection system. *ACTIN2* and *GAPDH* were used as internal controls in *Arabidopsis* and *Glycine soja*, respectively [76, 77]. cDNA quality was assessed by PCR using GADPH or ACTIN2 specific primers to exclude genomic DNA contamination. Expression levels for all genes were calculated using the 2^-ΔΔCT^ method [78]. Three independent biological replicates were carried out and subjected to enable statistical analysis. Primers efficiency was assessed by quantitative PCR and primers used for quantitative RT-PCR are listed in S1 Table.

### Subcellular localization

GsCML27-eGFP was constructed by digesting the coding region of *GsCML27* with *NcoI* and *SpeI* and cloning it into the pCAMBIA-1302 vector. Briefly, the full length *GsCML27* coding region was PCR amplified with the gene specific primer pair containing an *NcoI* site in the forward primer (5’-CATGCCATGGCCTTCTGTTGAAAGCGAT-3’) and a *SpeI* site in the reverse primer (5’-GGACTAGTCGGAGGATCTGTTTTTTCTTCAGCAT-3’). The PCR product was double-digested with *NcoI* and *SpeI*, and inserted into the *NcoI*/*SpeI* digested pCAMBIA-1302 vector, to generate pCAMBIA1302-GsCML27.

GsCML27-eGFP fusion protein and eGFP protein were transiently expressed in *Arabidopsis* protoplast cells as described [79]. Briefly, the rosette leaves of 3-week-old *Arabidopsis* were cut into 0.5–1 mm strips, and digested in 10 mL enzyme solution (20 mM MES (pH 5.7), 1.5% (w/v) cellulase R10, 0.4% (w/v) macerozyme R10, 0.4 M mannitol, 20 mM KCl, 10 mM CaCl_2_, 5 mM beta-mercaptoethanol, 0.1% (w/v) BSA) for 3 h at room temperature in dark. The solution was filtered with a nylon mesh after diluted with an equal volume of W5 solution (2 mM MES (pH 5.7), 150 mM NaCl, 125 mM CaCl_2_, 5 mM KCl). The protoplasts were centrifuged at 100 g for 2 min to pellet the protoplasts and re-suspended in W5 solution. Then, 100 μl of protoplasts, 10 μg of plasmid DNA and 110 μl of PEG solution (40% (w/v) PEG4000, 0.2 M mannitol, 100 mM CaCl_2_) were mixed completely, incubated at room temperature for 5–15 min, and wash with W5 solution twice. The transformed protoplasts were incubated for 10–16 h before checking the GFP signal by using a confocal laser-scanning microscope (SP2, Leica, Germany).

The plasmids eGFP and GsCML27-eGFP were precipitated onto gold beads to transient expression in onion epidermal cells. Localization of fluorescent protein in onion epidermal cells was observed at 488 nm using a confocal laser-scanning microscope (SP2, Leica, Germany). eGFP fluorescence and light field vision were recorded in separate channels and merged into an overlay image.

### Transformation of *Arabidopsis*

To identify the biological function of *GsCML27*, the coding region of *GsCML27* was cloned into the pCAMBIA330035S vector under the control of CaMV35S promoter through the USER^TM^ cloning technique [80]. Then the resulting pCAMBIA330035S:GsCML27 vector was introduced into *Agrobacterium tumefactions* strain LBA4404, and transformation of *Arabidopsis thaliana* was performed using the *Agrobacterium tumefactions* -mediated floral-dip method [81]. Transformants were selected on 1/2 MS medium containing 25 mg L^-1^ glufosinate ammonium, and the T_3_ generation from three independent ectopic expression transgenic lines (#13, #15, and #33) were randomly chosen for further functional studies.

### Phenotypic analysis of transgenic *Arabidopsis* under stress treatments

All *Arabidopsis* seeds were surfaced-sterilized as described [82]. During the seed germination and early seedling growth stage, the wild-type (WT) and ectopic expression seeds were sown on either normal 1/2 agar MS medium, or 1/2 MS medium, supplemented with either 10 mM NaHCO_3_, or 125 mM NaCl, or 300 mM mannitol, or KCl (50, 100, 150 mM) or LiCl (10, 20, 30 mM). The germination rates were recorded for consecutive 6 days after sowing. On the 7^th^ day, photos were taken to show the seedling growth performance, and the numbers of seedlings with open and green leaves were recorded.

For the root length assays, the 6-day-old WT and ectopic expression seedlings, grown on normal 1/2 MS medium, were transferred to fresh medium with 8 mM NaHCO_3_, 150 mM NaCl or 350 mM mannitol. The length of seedling primary roots was measured after vertical growth after 9 days. All experiments were repeated at least three times. The numerical data was subjected to statistical analyses using EXCEL and SPSS statistical softwares.

## Supporting Information

S1 Fig Transcriptome sequencing data of *GsCML27*. Transcriptome sequencing data of *GsCML27* in the wild type soybean *Glycine soja* G07256 under bicarbonate stress (50 mM NaHCO_3_, pH 8.5).
(TIF)

S2 Fig Ectopic expression of *GsCML27* in *Arabidopsis* did not response to KCl and LiCl during the seed germination stage. **A-G**. Growth performance of WT and ectopic expression seedlings on 1/2 MS medium with 50, 100, 150 mM KCl or 10, 20, 30 mM LiCl. Germination was recorded daily up to 5 days. Data shown represent the means (±SE) of three independent experiments.
(TIF)

S3 Fig Tissue specific expression of *GsCML27* in *Glycine soja* at early growth stage. Total RNA was extracted from *Glycine soja* at early growth stage (0 to 10 days). Relative expression levels were determined by quantitative RT-PCR using *GADPH* as an internal control. All of the values represent the means of three fully independent biological replicates.
(TIF)

S1 Table Gene-specific primers used for quantitative RT-PCR assays. (DOCX)

S2 Table Function of genes co-expression with *GsCML27*. (DOCX)

## References

[1] Tang C and Robson AD (1993) pH above 6.0 reduces nodulation in Lupinus species. Plant and Soil 152: 269–276.

[2] Yang C, Shi D and Wang D (2008) Comparative effects of salt and alkali stresses on growth, osmotic adjustment and ionic balance of an alkali-resistant halophyte Suaeda glauca (Bge.). Plant Growth Regulation 56: 179–190.

[3] Alhendawi RA, Römheld V, Kirkby EA and Marschner H (1997) Influence of increasing bicarbonate concentrations on plant growth, organic acid accumulation in roots and iron uptake by barley, sorghum, and maize. Journal of Plant Nutrition 20: 1731–1753.

[4] Jin H, Kim HR, Plaha P, Liu SK, Park JY, Piao YZ, et al (2008) Expression profiling of the genes induced by Na(2)CO(3) and NaCl stresses in leaves and roots of Leymus chinensis. Plant Science 175: 784–792.

[5] Zhao Y, Xu T, Shen CY, Xu GH, Chen SX, Song LZ, et al (2014) Identification of a retroelement from the resurrection plant Boea hygrometrica that confers osmotic and alkaline tolerance in Arabidopsis thaliana. PloS one 9: e98098 10.1371/journal.pone.0098098 24851859 PMC4031123

[6] Yang CP, Wang YC, Liu GF, Jiang J and Zhang GD (2005) [Study on expression of genes in Tamarix androssowii under NaHCO3 stress using gene chip technology]. Sheng wu gong cheng xue bao = Chinese journal of biotechnology 21: 220–226. 16013479

[7] Ge Y, Li Y, Zhu YM, Bai X, Lv DK, Guo D, et al (2010) Global transcriptome profiling of wild soybean (Glycine soja) roots under NaHCO3 treatment. BMC plant biology 10: 153 10.1186/1471-2229-10-153 20653984 PMC3017823

[8] Gong B, Zhang C, Li X, Wen D, Wang S, Shi Q, et al (2014) Identification of NaCl and NaHCO3 stress responsive proteins in tomato roots using iTRAQ-based analysis. Biochemical and biophysical research communications 446: 417–422. 10.1016/j.bbrc.2014.03.005 24613841

[9] Zhu D, Bai X, Chen C, Chen Q, Cai H, Li Y, et al (2011) GsTIFY10, a novel positive regulator of plant tolerance to bicarbonate stress and a repressor of jasmonate signaling. Plant molecular biology 77: 285–297. 10.1007/s11103-011-9810-0 21805375

[10] Liu L, Wang Y, Wang N, Dong YY, Fan XD, Liu XM, et al (2011) Cloning of a vacuolar H(+)-pyrophosphatase gene from the halophyte Suaeda corniculata whose heterologous overexpression improves salt, saline-alkali and drought tolerance in Arabidopsis. Journal of integrative plant biology 53: 731–742. 10.1111/j.1744-7909.2011.01066.x 21762382

[11] Ishitani M, Liu J, Halfter U, Kim CS, Shi W and Zhu JK (2000) SOS3 function in plant salt tolerance requires N-myristoylation and calcium binding. The Plant cell 12: 1667–1678. 11006339 10.1105/tpc.12.9.1667 PMC149077

[12] Liu J, Ishitani M, Halfter U, Kim CS and Zhu JK (2000) The Arabidopsis thaliana SOS2 gene encodes a protein kinase that is required for salt tolerance. Proceedings of the National Academy of Sciences of the United States of America 97: 3730–3734. 10725382 10.1073/pnas.060034197 PMC16308

[13] Shi H, Ishitani M, Kim C and Zhu JK (2000) The Arabidopsis thaliana salt tolerance gene SOS1 encodes a putative Na+/H+ antiporter. Proceedings of the National Academy of Sciences of the United States of America 97: 6896–6901. 10823923 10.1073/pnas.120170197 PMC18772

[14] Xiong L, Schumaker KS and Zhu JK (2002) Cell signaling during cold, drought, and salt stress. The Plant cell 14 Suppl: S165–183. 12045276 10.1105/tpc.000596 PMC151254

[15] Zhu JK (2002) Salt and drought stress signal transduction in plants. Annual review of plant biology 53: 247–273. 12221975 10.1146/annurev.arplant.53.091401.143329 PMC3128348

[16] Zhu JK (2001) Plant salt tolerance. Trends in plant science 6: 66–71. 11173290 10.1016/s1360-1385(00)01838-0

[17] Chinnusamy V, Schumaker K and Zhu JK (2004) Molecular genetic perspectives on cross-talk and specificity in abiotic stress signalling in plants. Journal of experimental botany 55: 225–236. 14673035 10.1093/jxb/erh005

[18] Huang GT, Ma SL, Bai LP, Zhang L, Ma H, Jia P, et al (2012) Signal transduction during cold, salt, and drought stresses in plants. Molecular biology reports 39: 969–987. 10.1007/s11033-011-0823-1 21573796

[19] Zhang J, Zou D, Li Y, Sun X, Wang NN, Gong SY, et al (2014) GhMPK17, a cotton mitogen-activated protein kinase, is involved in plant response to high salinity and osmotic stresses and ABA signaling. PloS one 9: e95642 10.1371/journal.pone.0095642 24743296 PMC3990703

[20] Xiong L and Zhu JK (2001) Abiotic stress signal transduction in plants: Molecular and genetic perspectives. Physiologia plantarum 112: 152–166. 11454221 10.1034/j.1399-3054.2001.1120202.x

[21] Takahashi S, Katagiri T, Hirayama T, Yamaguchi-Shinozaki K and Shinozaki K (2001) Hyperosmotic stress induces a rapid and transient increase in inositol 1,4,5-trisphosphate independent of abscisic acid in Arabidopsis cell culture. Plant & cell physiology 42: 214–222. 11230576 10.1093/pcp/pce028

[22] Harshavardhan VT, Van Son L, Seiler C, Junker A, Weigelt-Fischer K, Klukas C, et al (2014) AtRD22 and AtUSPL1, members of the plant-specific BURP domain family involved in Arabidopsis thaliana drought tolerance. PloS one 9: e110065 10.1371/journal.pone.0110065 25333723 PMC4198191

[23] Yang CW, Xu HH, Wang LL, Liu J, Shi DC and Wang DL (2009) Comparative effects of salt-stress and alkali-stress on the growth, photosynthesis, solute accumulation, and ion balance of barley plants. Photosynthetica 47: 79–86.

[24] Jiang Z, Zhu S, Ye R, Xue Y, Chen A, An L, et al (2013) Relationship between NaCl- and H2O2-induced cytosolic Ca2+ increases in response to stress in Arabidopsis. PloS one 8: e76130 10.1371/journal.pone.0076130 24124535 PMC3790670

[25] Takahashi S, Katagiri T, Yamaguchi-Shinozaki K and Shinozaki K (2000) An Arabidopsis gene encoding a Ca2+-binding protein is induced by abscisic acid during dehydration. Plant & cell physiology 41: 898–903. 10965948 10.1093/pcp/pcd010

[26] Gao D, Knight MR, Trewavas AJ, Sattelmacher B and Plieth C (2004) Self-reporting Arabidopsis expressing pH and [Ca2+] indicators unveil ion dynamics in the cytoplasm and in the apoplast under abiotic stress. Plant physiology 134: 898–908. 15020753 10.1104/pp.103.032508 PMC389913

[27] Gong D, Guo Y, Schumaker KS and Zhu JK (2004) The SOS3 family of calcium sensors and SOS2 family of protein kinases in Arabidopsis. Plant physiology 134: 919–926. 15020756 10.1104/pp.103.037440 PMC523892

[28] Kolukisaoglu U, Weinl S, Blazevic D, Batistic O and Kudla J (2004) Calcium sensors and their interacting protein kinases: genomics of the Arabidopsis and rice CBL-CIPK signaling networks. Plant physiology 134: 43–58. 14730064 10.1104/pp.103.033068 PMC316286

[29] Kim KN, Cheong YH, Gupta R and Luan S (2000) Interaction specificity of Arabidopsis calcineurin B-like calcium sensors and their target kinases. Plant physiology 124: 1844–1853. 11115898 10.1104/pp.124.4.1844 PMC59879

[30] Hashimoto K and Kudla J (2011) Calcium decoding mechanisms in plants. Biochimie 93: 2054–2059. 10.1016/j.biochi.2011.05.019 21658427

[31] Reddy AS (2001) Calcium: silver bullet in signaling. Plant science: an international journal of experimental plant biology 160: 381–404. 11166425 10.1016/s0168-9452(00)00386-1

[32] Kretsinger RH and Nockolds CE (1973) Carp muscle calcium-binding protein. II. Structure determination and general description. The Journal of biological chemistry 248: 3313–3326. 4700463

[33] McCormack E and Braam J (2003) Calmodulins and related potential calcium sensors of Arabidopsis. New Phytologist 159: 585–598. 10.1046/j.1469-8137.2003.00845.x 33873603

[34] McCormack E, Tsai YC and Braam J (2005) Handling calcium signaling: Arabidopsis CaMs and CMLs. Trends in plant science 10: 383–389. 16023399 10.1016/j.tplants.2005.07.001

[35] Delk NA, Johnson KA, Chowdhury NI and Braam J (2005) CML24, regulated in expression by diverse stimuli, encodes a potential Ca2+ sensor that functions in responses to abscisic acid, daylength, and ion stress. Plant Physiol 139: 240–253. 16113225 10.1104/pp.105.062612 PMC1203374

[36] Yamaguchi T, Aharon GS, Sottosanto JB and Blumwald E (2005) Vacuolar Na+/H+ antiporter cation selectivity is regulated by calmodulin from within the vacuole in a Ca2+- and pH-dependent manner. Proceedings of the National Academy of Sciences of the United States of America 102: 16107–16112. 16249341 10.1073/pnas.0504437102 PMC1276053

[37] Magnan F, Ranty B, Charpenteau M, Sotta B, Galaud JP and Aldon D (2008) Mutations in AtCML9, a calmodulin-like protein from Arabidopsis thaliana, alter plant responses to abiotic stress and abscisic acid. Plant J 56: 575–589. 10.1111/j.1365-313X.2008.03622.x 18643966

[38] Vanderbeld B and Snedden WA (2007) Developmental and stimulus-induced expression patterns of Arabidopsis calmodulin-like genes CML37, CML38 and CML39. Plant molecular biology 64: 683–697. 17579812 10.1007/s11103-007-9189-0

[39] Ge Y, Zhu Y, Lv D, Dong T, Wang W, Tan S, et al (2009) Research on responses of wild soybean to alkaline stress. Pratacultural Science 26: 47–52.

[40] DuanMu H, Wang Y, Bai X, Cheng S, Deyholos MK, Wong GK, et al (2015) Wild soybean roots depend on specific transcription factors and oxidation reduction related genesin response to alkaline stress. Functional & integrative genomics. 10.1007/s10142-015-0439-y 25874911

[41] Day IS, Reddy VS, Shad Ali G and Reddy AS (2002) Analysis of EF-hand-containing proteins in Arabidopsis. Genome biology 3: RESEARCH0056 12372144 10.1186/gb-2002-3-10-research0056 PMC134623

[42] Grabarek Z (2006) Structural basis for diversity of the EF-hand calcium-binding proteins. Journal of molecular biology 359: 509–525. 16678204 10.1016/j.jmb.2006.03.066

[43] Herzberg O, Moult J and James MN (1986) A model for the Ca2+-induced conformational transition of troponin C. A trigger for muscle contraction. The Journal of biological chemistry 261: 2638–2644. 3949740

[44] Wang Z, Gergely J and Tao T (1992) Characterization of the Ca(2+)-triggered conformational transition in troponin C. Proceedings of the National Academy of Sciences of the United States of America 89: 11814–11817. 1465405 10.1073/pnas.89.24.11814 PMC50647

[45] Shi DC and Sheng YM (2005) Effect of various salt-alkaline mixed stress conditions on sunflower seedlings and analysis of their stress factors. Environ Exp Bot 54: 8–21.

[46] Yang CW, Chong JN, Li CY, Kim CM, Shi DC and Wang DL (2007) Osmotic adjustment and ion balance traits of an alkali resistant halophyte Kochia sieversiana during adaptation to salt and alkali conditions. Plant Soil 294: 263–276.

[47] Zhu J-K, Hasegawa PM, Bressan RA and Bohnert HJ (1997) Molecular aspects of osmotic stress in plants. Critical Reviews in Plant Sciences 16: 253–277.

[48] DeFalco TA, Bender KW and Snedden WA (2010) Breaking the code: Ca2+ sensors in plant signalling. Biochem J 425: 27–40. 10.1042/BJ20091147 20001960

[49] Niki I, Yokokura H, Sudo T, Kato M and Hidaka H (1996) Ca2+ signaling and intracellular Ca2+ binding proteins. Journal of biochemistry 120: 685–698. 8947828 10.1093/oxfordjournals.jbchem.a021466

[50] Dobney S, Chiasson D, Lam P, Smith SP and Snedden WA (2009) The Calmodulin-related Calcium Sensor CML42 Plays a Role in Trichome Branching. Journal of Biological Chemistry 284: 31647–31657. 10.1074/jbc.M109.056770 19720824 PMC2797235

[51] Magnan F, Ranty B, Charpenteau M, Sotta B, Galaud JP and Aldon D (2008) Mutations in AtCML9, a calmodulin-like protein from Arabidopsis thaliana, alter plant responses to abiotic stress and abscisic acid. The Plant journal: for cell and molecular biology 56: 575–589. 18643966 10.1111/j.1365-313X.2008.03622.x

[52] Zhu D, Bai X, Chen C, Chen Q, Cai H, Li Y, et al (2011) GsTIFY10, a novel positive regulator of plant tolerance to bicarbonate stress and a repressor of jasmonate signaling. Plant molecular biology 77: 285–297. 10.1007/s11103-011-9810-0 21805375

[53] Zhu D, Li RT, Liu X, Sun MZ, Wu J, Zhang N, et al (2014) The Positive Regulatory Roles of the TIFY10 Proteins in Plant Responses to Alkaline Stress. PloS one 9. 10.1371/journal.pone.0111984 PMC4222965 25375909

[54] Liu X, Song Y, Xing F, Wang N, Wen F and Zhu C (2015) GhWRKY25, a group I WRKY gene from cotton, confers differential tolerance to abiotic and biotic stresses in transgenic Nicotiana benthamiana. Protoplasma. 26410829 10.1007/s00709-015-0885-3

[55] Jiang YQ and Deyholos MK (2009) Functional characterization of Arabidopsis NaCl-inducible WRKY25 and WRKY33 transcription factors in abiotic stresses. Plant molecular biology 69: 91–105. 10.1007/s11103-008-9408-3 18839316

[56] Park HC, Park CY, Koo SC, Cheong MS, Kim KE, Kim MC, et al (2010) AtCML8, a calmodulin-like protein, differentially activating CaM-dependent enzymes in Arabidopsis thaliana. Plant cell reports 29: 1297–1304. 10.1007/s00299-010-0916-7 20820784

[57] Delk NA, Johnson KA, Chowdhury NI and Braam J (2005) CML24, regulated in expression by diverse stimuli, encodes a potential Ca2+ sensor that functions in responses to abscisic acid, daylength, and ion stress. Plant physiology 139: 240–253. 16113225 10.1104/pp.105.062612 PMC1203374

[58] Vadassery J, Reichelt M, Hause B, Gershenzon J, Boland W and Mithofer A (2012) CML42-Mediated Calcium Signaling Coordinates Responses to Spodoptera Herbivory and Abiotic Stresses in Arabidopsis. Plant physiology 159: 1159–1175. 10.1104/pp.112.198150 22570470 PMC3387702

[59] Perochon A, Aldon D, Galaud JP and Ranty B (2011) Calmodulin and calmodulin-like proteins in plant calcium signaling. Biochimie 93: 2048–2053. 10.1016/j.biochi.2011.07.012 21798306

[60] Vanderbeld B and Snedden WA (2007) Developmental and stimulus-induced expression patterns of Arabidopsis calmodulin-like genes CML37, CML38 and CML39. Plant molecular biology 64: 683–697. 17579812 10.1007/s11103-007-9189-0

[61] Zhu D, Cai H, Luo X, Bai X, Deyholos MK, Chen Q, et al (2012) Over-expression of a novel JAZ family gene from Glycine soja, increases salt and alkali stress tolerance. Biochemical and biophysical research communications 426: 273–279. 10.1016/j.bbrc.2012.08.086 22943855

[62] Zhu D, Li R, Liu X, Sun M, Wu J, Zhang N, et al (2014) The positive regulatory roles of the TIFY10 proteins in plant responses to alkaline stress. PloS one 9: e111984 10.1371/journal.pone.0111984 25375909 PMC4222965

[63] Perruc E, Charpenteau M, Ramirez BC, Jauneau A, Galaud JP, Ranjeva R, et al (2004) A novel calmodulin-binding protein functions as a negative regulator of osmotic stress tolerance in Arabidopsis thaliana seedlings. Plant J 38: 410–420. 15086802 10.1111/j.1365-313X.2004.02062.x

[64] Jung C, Seo JS, Han SW, Koo YJ, Kim CH, Song SI, et al (2008) Overexpression of AtMYB44 enhances stomatal closure to confer abiotic stress tolerance in transgenic Arabidopsis. Plant physiology 146: 623–635. 18162593 10.1104/pp.107.110981 PMC2245844

[65] Soyka S and Heyer AG (1999) Arabidopsis knockout mutation of ADC2 gene reveals inducibility by osmotic stress. FEBS letters 458: 219–223. 10481069 10.1016/s0014-5793(99)01125-4

[66] Cheong YH, Kim KN, Pandey GK, Gupta R, Grant JJ and Luan S (2003) CBL1, a calcium sensor that differentially regulates salt, drought, and cold responses in Arabidopsis. The Plant cell 15: 1833–1845. 12897256 10.1105/tpc.012393 PMC167173

[67] Knight H and Knight MR (2001) Abiotic stress signalling pathways: specificity and cross-talk. Trends in plant science 6: 262–267. 11378468 10.1016/s1360-1385(01)01946-x

[68] Mahajan S and Tuteja N (2005) Cold, salinity and drought stresses: an overview. Archives of biochemistry and biophysics 444: 139–158. 16309626 10.1016/j.abb.2005.10.018

[69] Tuteja N (2007) Mechanisms of high salinity tolerance in plants. Methods in enzymology 428: 419–438. 17875432 10.1016/S0076-6879(07)28024-3

[70] Chen S and Polle A (2010) Salinity tolerance of Populus. Plant biology 12: 317–333. 10.1111/j.1438-8677.2009.00301.x 20398238

[71] Yan A, Wu MJ, Yan LM, Hu R, Ali I and Gan YB (2014) AtEXP2 Is Involved in Seed Germination and Abiotic Stress Response in Arabidopsis. PloS one 9. 10.1371/journal.pone.0085208 PMC3880340 24404203

[72] Scarpeci TE, Zanor MI, Mueller-Roeber B and Valle EM (2013) Overexpression of AtWRKY30 enhances abiotic stress tolerance during early growth stages in Arabidopsis thaliana. Plant molecular biology 83: 265–277. 10.1007/s11103-013-0090-8 23794142

[73] Kumar S, Nei M, Dudley J and Tamura K (2008) MEGA: a biologist-centric software for evolutionary analysis of DNA and protein sequences. Briefings in bioinformatics 9: 299–306. 10.1093/bib/bbn017 18417537 PMC2562624

[74] Singh R, Hassan MI, Islam A and Ahmad F (2015) Cooperative Unfolding of Residual Structure in Heat Denatured Proteins by Urea and Guanidinium Chloride. PloS one 10. 10.1371/journal.pone.0128740 PMC4457810 26046628

[75] Correia M, Snabe T, Thiagarajan V, Petersen SB, Campos SR, Baptista AM, et al (2015) Photonic activation of plasminogen induced by low dose UVB. PloS one 10: e0116737 10.1371/journal.pone.0116737 25635856 PMC4312030

[76] Huis R, Hawkins S and Neutelings G (2010) Selection of reference genes for quantitative gene expression normalization in flax (Linum usitatissimum L.). BMC plant biology 10. 10.1186/1471-2229-10-71 PMC3095345 20403198

[77] Czechowski T, Stitt M, Altmann T, Udvardi MK and Scheible WR (2005) Genome-wide identification and testing of superior reference genes for transcript normalization in Arabidopsis. Plant physiology 139: 5–17. 16166256 10.1104/pp.105.063743 PMC1203353

[78] Livak KJ and Schmittgen TD (2001) Analysis of relative gene expression data using real-time quantitative PCR and the 2(-Delta Delta C(T)) Method. Methods 25: 402–408. 11846609 10.1006/meth.2001.1262

[79] Yoo SD, Cho YH and Sheen J (2007) Arabidopsis mesophyll protoplasts: a versatile cell system for transient gene expression analysis. Nature protocols 2: 1565–1572. 17585298 10.1038/nprot.2007.199

[80] Nour-Eldin HH, Hansen BG, Norholm MH, Jensen JK and Halkier BA (2006) Advancing uracil-excision based cloning towards an ideal technique for cloning PCR fragments. Nucleic acids research 34: e122 17000637 10.1093/nar/gkl635 PMC1635280

[81] Clough SJ and Bent AF (1998) Floral dip: a simplified method for Agrobacterium-mediated transformation of Arabidopsis thaliana. Plant J 16: 735–743. 10069079 10.1046/j.1365-313x.1998.00343.x

[82] Sun XL, Ji W, Ding XD, Bai X, Cai H, Yang SS, et al (2013) GsVAMP72, a novel Glycine soja R-SNARE protein, is involved in regulating plant salt tolerance and ABA sensitivity. Plant Cell Tiss Org 113: 199–215.

