# Supplementary figures and images for: GsCML27, a Gene Encoding a Calcium-Binding Ef-Hand Protein from Glycine soja, Plays Differential Roles in Plant Responses to Bicarbonate, Salt and Osmotic Stresses
Source: PLoS One. 2015 Nov 9;10(11):e0141888. doi: 10.1371/journal.pone.0141888 (PMC4638360; doi:10.1371/journal.pone.0141888)

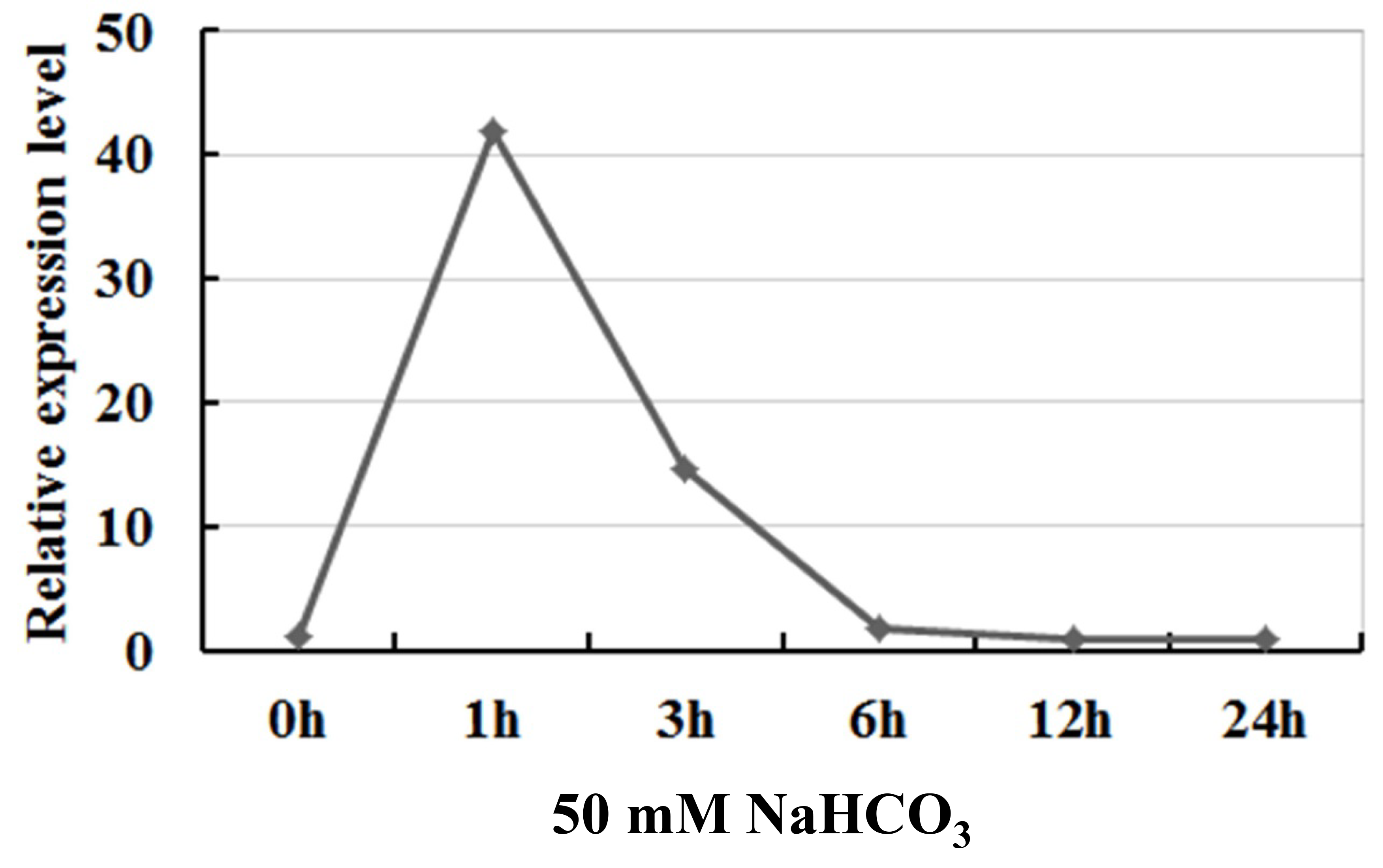

Supplement: S1 Fig — Transcriptome sequencing data of GsCML27 in the wild type soybean Glycine soja G07256 under bicarbonate stress (50 mM NaHCO3, pH 8.5). (TIF) [file pone.0141888.s001.tif]

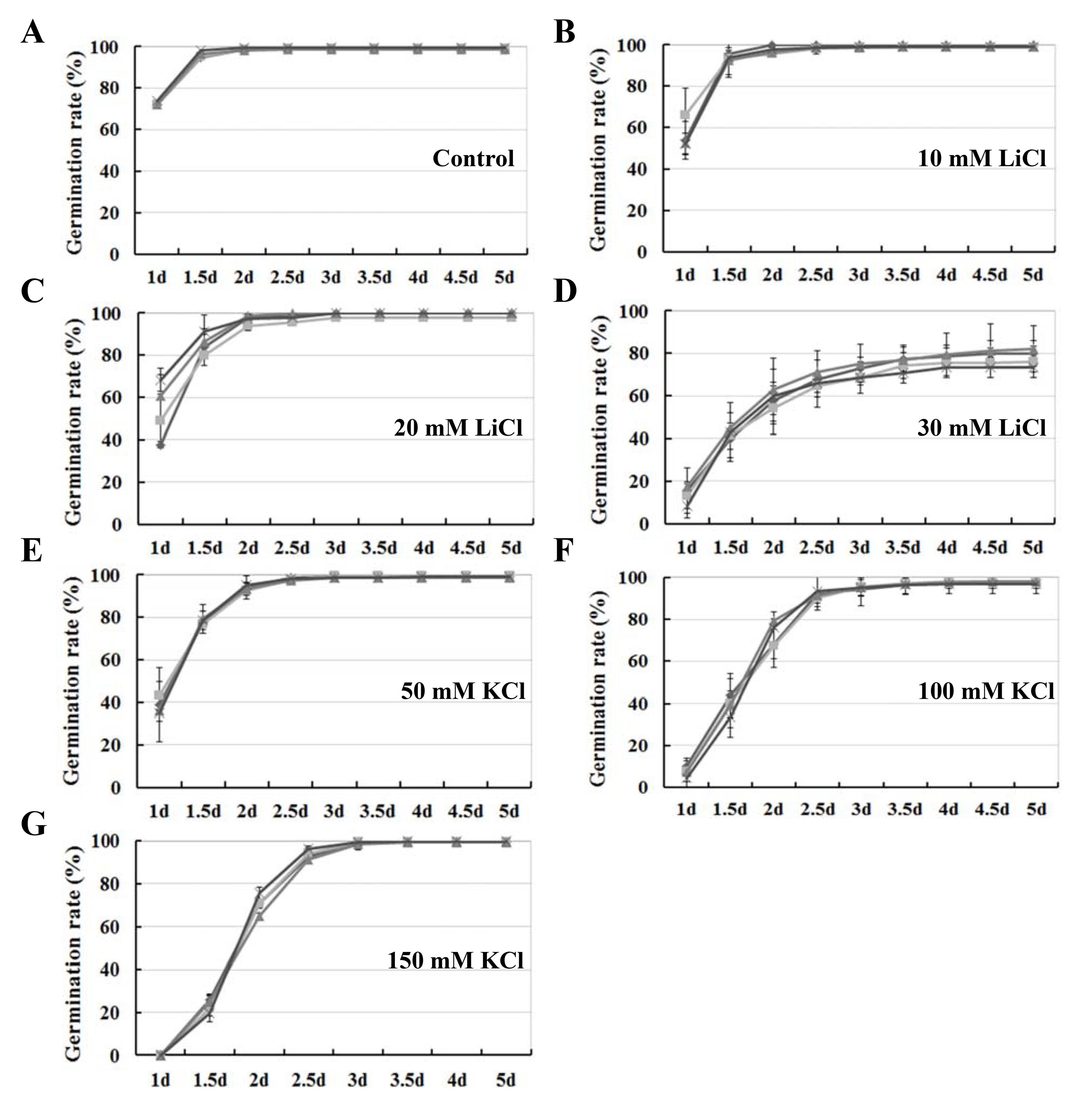

Supplement: S2 Fig — A-G. Growth performance of WT and ectopic expression seedlings on 1/2 MS medium with 50, 100, 150 mM KCl or 10, 20, 30 mM LiCl. Germination was recorded daily up to 5 days. Data shown represent the means (±SE) of three independent experiments. (TIF) [file pone.0141888.s002.tif]

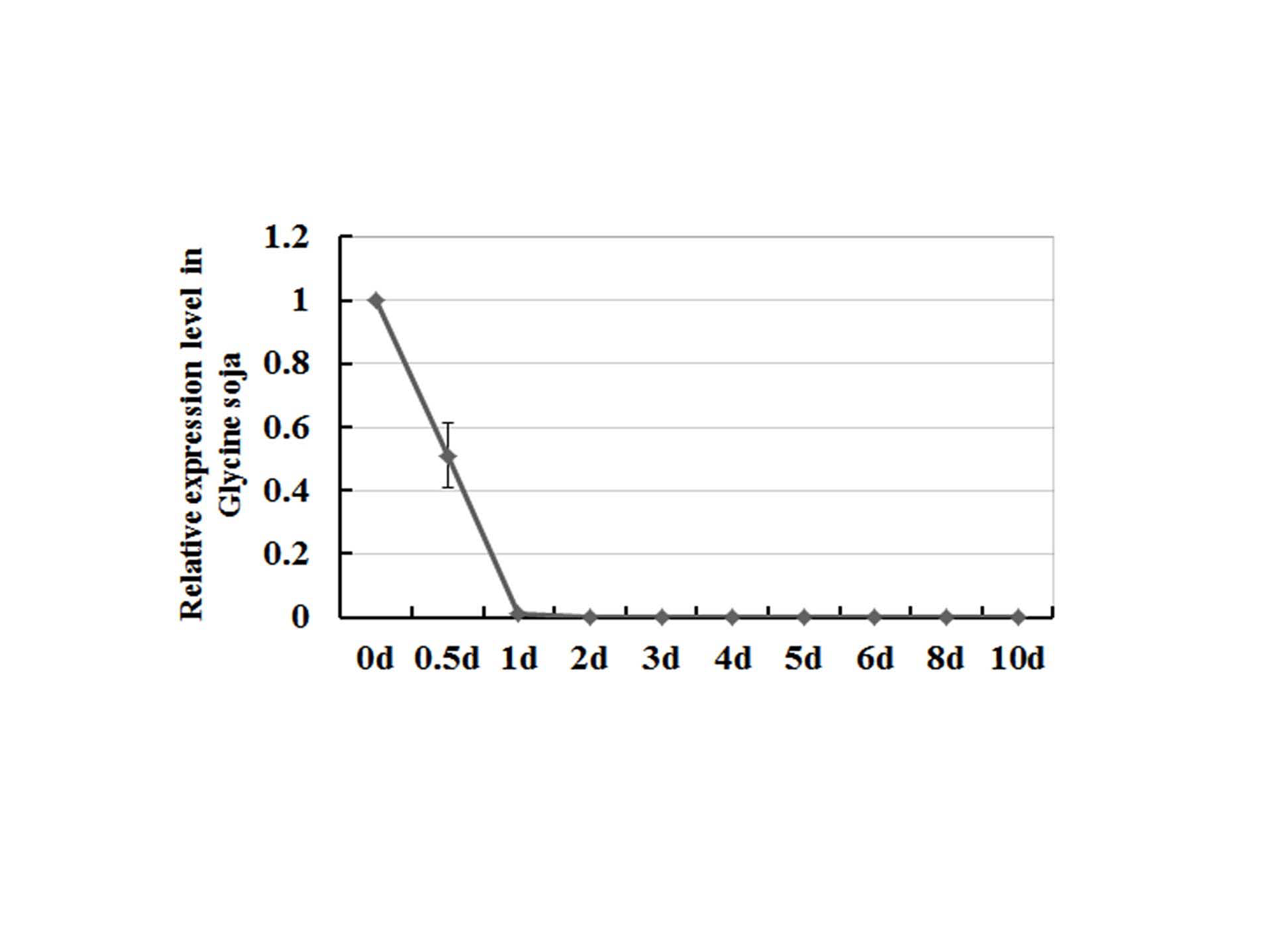

Supplement: S3 Fig — Total RNA was extracted from Glycine soja at early growth stage (0 to 10 days). Relative expression levels were determined by quantitative RT-PCR using GADPH as an internal control. All of the values represent the means of three fully independent biological replicates. (TIF) [file pone.0141888.s003.tif]
