# Supplementary material for: GsCML27, a Gene Encoding a Calcium-Binding Ef-Hand Protein from Glycine soja, Plays Differential Roles in Plant Responses to Bicarbonate, Salt and Osmotic Stresses
Source: PLoS One. 2015 Nov 9;10(11):e0141888. doi: 10.1371/journal.pone.0141888 (PMC4638360; doi:10.1371/journal.pone.0141888)
Supplement: S1 Table — (DOCX) [file pone.0141888.s004.docx]

**S1 Table. Gene-specific primers used for quantitative RT-PCR assays.**

| **Gene name** | **Primer sequence (5’-3’)** |
| --- | --- |
| *GsCML27* | Forward: CCAAATCCTCCGTGTACCTGC |
|  | Reverse: GAGGTTAATGAAGCCGTCGTG |
| *GAPDH* | Forward: GACTGGTATGGCATTCCGTGT |
|  | Reverse: GCCCTCTGATTCCTCCTTGA |
| *ACTIN2* | Forward: TTACCCGATGGGCAAGTC |
|  | Reverse: GCTCATACGGTCAGCGATAC |
| *P5CS* | Forward: CAAGATGAGATTACATTCGGCG |
|  | Reverse: TACCCACTGGTTATGATGACAGG |
| *RD22* | Forward: TACCAAACACTCCCATTCCCA |
|  | Reverse: GCGTACACCTCCCTTTCCAAC |
| *COR47* | Forward: GGAGTACAAGAACAACGTTCCCGA |
|  | Reverse: TGTCGTCGCTGGTGATTCCTCT |
