# Supplementary material for: GsCML27, a Gene Encoding a Calcium-Binding Ef-Hand Protein from Glycine soja, Plays Differential Roles in Plant Responses to Bicarbonate, Salt and Osmotic Stresses
Source: PLoS One. 2015 Nov 9;10(11):e0141888. doi: 10.1371/journal.pone.0141888 (PMC4638360; doi:10.1371/journal.pone.0141888)
Supplement: S2 Table — (DOCX) [file pone.0141888.s005.docx]

**S2 Table. Function of genes co-expression with *GsCML27*.**

| **Stress** | **Locus tag (*Soybean*)** | **Locus tag (*Arabidopsis*)** | **Annotated** |
| --- | --- | --- | --- |
| **Alkali** | Glyma01g41290 (*GsJAZ2*) | At1g74950 | protein TIFY 10B |
|  | Glyma11g04130 (*GsTIFY10a*) | At1g19180 | jasmonate-zim-domain protein 1 |
|  | Glyma07g04630 (*GsTIFY11b*) | At1g72450 | jasmonate-zim-domain protein 6 |
|  | Glyma16g01220 (*GsTIFY11b*) | At1g72450 | jasmonate-zim-domain protein 6 |
| **Drought, Salt** | Glyma03g27560 | At2g41010 (*AtCAMBP25*) | calmodulin binding protein 25 |
|  | Glyma17g35620 | At5g67300 (*AtMYB44*) | transcription factor MYB44 |
|  | Glyma07g36430 | At5g49620 (*AtMYB78*) | myb domain protein 78 |
|  | Glyma10g40890 | At2g02360 | F-box protein PP2-B10 |
|  | Glyma19g32260 | At5g19450 | calcium-dependent protein kinase 19 |
|  | Glyma06g00990 | At4g34710 (*AtADC2*) | arginine decarboxylase 2 |
|  | Glyma20g26940 | At1g27730 (*ZAT10*) | zinc finger protein STZ/ZAT10 |
|  | Glyma11g11430 | At2g17840 (*ERD7*) | senescence/dehydration related protein |
|  | Glyma19g38670 | At3g52450 (*PUB22*) | E3 ubiquitin-protein ligase PUB22 |
|  | Glyma19g38740 | At3g52450 (*PUB22*) | E3 ubiquitin-protein ligase PUB22 |

Co-expression between genes were evaluated by Pearson Correlation Coefficient (PCC) with the threshold value: PCC>0.9, p<0.05. Locus tags were confirmed through Phytozome (http://www.phytozome.org/), and function of co-expression genes were annotated by NCBI (http://www.ncbi.nlm.nih.gov/).
